# Lessons learned in a data linkage project on fatal drowning

**DOI:** 10.1186/s40621-026-00660-x

**Published:** 2026-02-01

**Authors:** Rohit P. Shenoi, Ned Levine, Elizabeth A. Camp, Linh Nguyen, Sandra McKay, Shaila Zaman

**Affiliations:** 1 Division of Emergency Medicine, Department of Pediatrics, Texas Children’s Hospital and Baylor College of Medicine, 6621 Fannin St., Suite A 2210, Houston, Texas, 77030 USA; 2 Ned Levine and Associates, Houston, Texas, 77025 USA; 3 Division of Emergency Medicine, Department of Pediatrics, Texas Children’s Hospital and Baylor College of Medicine, 6621 Fannin St., Suite A 2210, Houston, Texas, 77030 USA; 4 Center for Population Health Management & Quality, Department of Healthcare Transformation Initiatives, The University of Texas Health Science Center at Houston, Houston, Texas, 77030 USA; 5 Department of Pediatrics at McGovern Medical School, The University of Texas Health Science Center at Houston, Houston, Texas, 77030 USA; 6 Division of Emergency Medicine, Department of Pediatrics, Texas Children’s Hospital and Baylor College of Medicine, 6621 Fannin St., Suite A 2210, Houston, Texas, 77030 USA

**Keywords:** Probabilistic data linkage, Drowning, Lessons learned, Epidemiology

## Abstract

**Background:**

Drowning is the leading cause of death in US children 1–4 years old. The epidemiology of drowning at a regional level is understudied because no single data source provides complete information on persons who drown. Probabilistic data linkage is a novel way of studying the epidemiology of drowning. This study aimed to document the lessons learned during the linkage process.

**Methods:**

This was a cross-sectional study of persons of all ages who died from unintentional drowning in metropolitan Houston from 2016 to 2022. We describe the lessons learned during the project planning and execution phases which pertained to data curation, the regulatory aspects involved with obtaining data, data security, spatial identification, and the strengths and limitations of the different datasets.

**Results:**

Twelve datasets were reviewed; eight were successfully linked. During the planning phase, the key issues identified pertained to data ownership and governance and robustness of data which impacted the availability and quality of data, variation in the description of drowning location, and risk and protective factors which helped identify subpopulations at-risk for drowning. In the execution phase, the major issues included data security, data sharing, and dissemination of results.

**Conclusion:**

There are a plethora of data sources for fatal drowning. The process of obtaining and analyzing data to describe the epidemiology of fatal drowning using probabilistic data linkage is complex, lengthy, and cumbersome. Documenting the process and lessons learned can support drowning research and inform regional drowning prevention strategies.

**Supplementary Information:**

The online version contains supplementary material available at 10.1186/s40621-026-00660-x.

## Background

Drowning is one of the top three causes of death by unintentional injury worldwide [1]. In the United States, highest unintentional drowning fatality rates occur in children 1–4 years old [2] and adults aged ≥ 65 years [3]. Death certificate data have been used to describe the injury burden and demographics of fatal drowning. Syndromic surveillance data can describe the epidemiology of fatal and non-fatal drowning in real time among persons who seek medical care [4–6]. However, these surveillance methods do not provide information on the location, circumstances, risk, and protective factors associated with drowning events. Other limitations of syndromic surveillance include inconsistent characterization of the body of water and drowning outcome. In-depth death investigations and the narratives in forensic reports of drowning fatalities provide a rich source of information on the circumstances, risk and protective factors that existed at the time of drowning. The child death review form captures rich information of the circumstances of fatal drowning. However, not all states utilize this resource [7]. Countries such as Australia and New Zealand utilize more robust databases such as the National Coronial Information System (NCIS) (https://www.ncis.org.au). NCIS records and provides public health research access to comprehensive fatal injury data, including drowning. These data are available for whole populations or discrete geographic areas [8, 9].

Probabilistic linkage of data from multiple sources that describe drowning events, such as medical examiner, hospital, emergency medical services (EMS), environmental agencies, maritime organizations, and media reports, is a novel method for studying the epidemiology, risk and protective factors, and locations of fatal unintentional drowning at the regional level [10]. This information is vital when instituting countermeasures at the local level. The data sources and linkage methodology are described elsewhere [10]. The goal of this paper is to describe the lessons learned during the probabilistic linkage project pertaining to data curation, the regulatory aspects involved with obtaining data, documenting variables, data security, and the strengths and limitations of the different datasets that were used during data linkage. The benefits and drawbacks of probabilistic data linkage are not discussed since they are explained in the original study [10]. 

## Methods

This was a cross-sectional study of unintentional fatal drowning in persons of all ages that occurred in metropolitan Houston between 2016 and 2022. Persons who unintentionally drowned in constructed bodies of water (swimming pools, hot tubs, bathtubs), natural water (beach, bay, lake, river), flood control structures (bayou, canal, creek, ditch, concrete waterway, reservoir, pond, retention pond), or flooding events were included. Drownings due to intentional or undetermined causes were excluded. The metropolitan Houston region comprises eight Texas counties: Brazoria, Chambers, Galveston, Fort Bend, Harris (which includes the City of Houston), Liberty, Montgomery, and Waller. The area encompasses the Texas Gulf coast and the vast Galveston Bay, four large lakes, three river systems, and numerous estuaries, bays, and inlets. There are also numerous flood control structures such as retention ponds, canals, and waterways. In 2022, the end date for our study, the population of the region was 7,092,073 and there were approximately 191,578 permanent swimming pools. Children constituted 26% of the population. By race and ethnicity, the population consisted of non-Hispanic White (34%), African-American (19%), Asian (8%), American Indian/Alaskan Native (1%), and Hispanic (39%) persons [11]. 

We used the methodology described by Dusetzina et al. [12] for the linkage project. The project consisted of a planning phase and an execution phase. The project planning phase consisted of feasibility assessment, data ownership and governance, technical environment, team skills and expertise, and cost of data acquisition and analysis. In the project execution phase, the topics included data partnerships, data use agreements, spatial geocoding, and probabilistic linkage. The methodology of probabilistic linkage, i.e., identifying and evaluating identifiers/linkage variables, standardizing, and cleaning linkage variables, developing an analysis database, determining the linkage approach, evaluation, and validation of linkage, and reporting of linkage metrics were described in the original study [10]. 

This study received institutional review board (IRB) approval from the Baylor College of Medicine (H-51536), University of Texas at Houston (HSC-MS-22–0427) and Department of State Health Services Texas (IRB# 24 − 020).

### (A) project planning

#### Feasibility assessment

*Data Sources*: It was important to assess whether the linkage process was feasible and would result in a meaningful, enriched linked dataset. This involved evaluating the quality and robustness of linkage identifiers as well as the degree of overlap among sub-populations represented across various datasets [12]. 

**Fig. 1:**
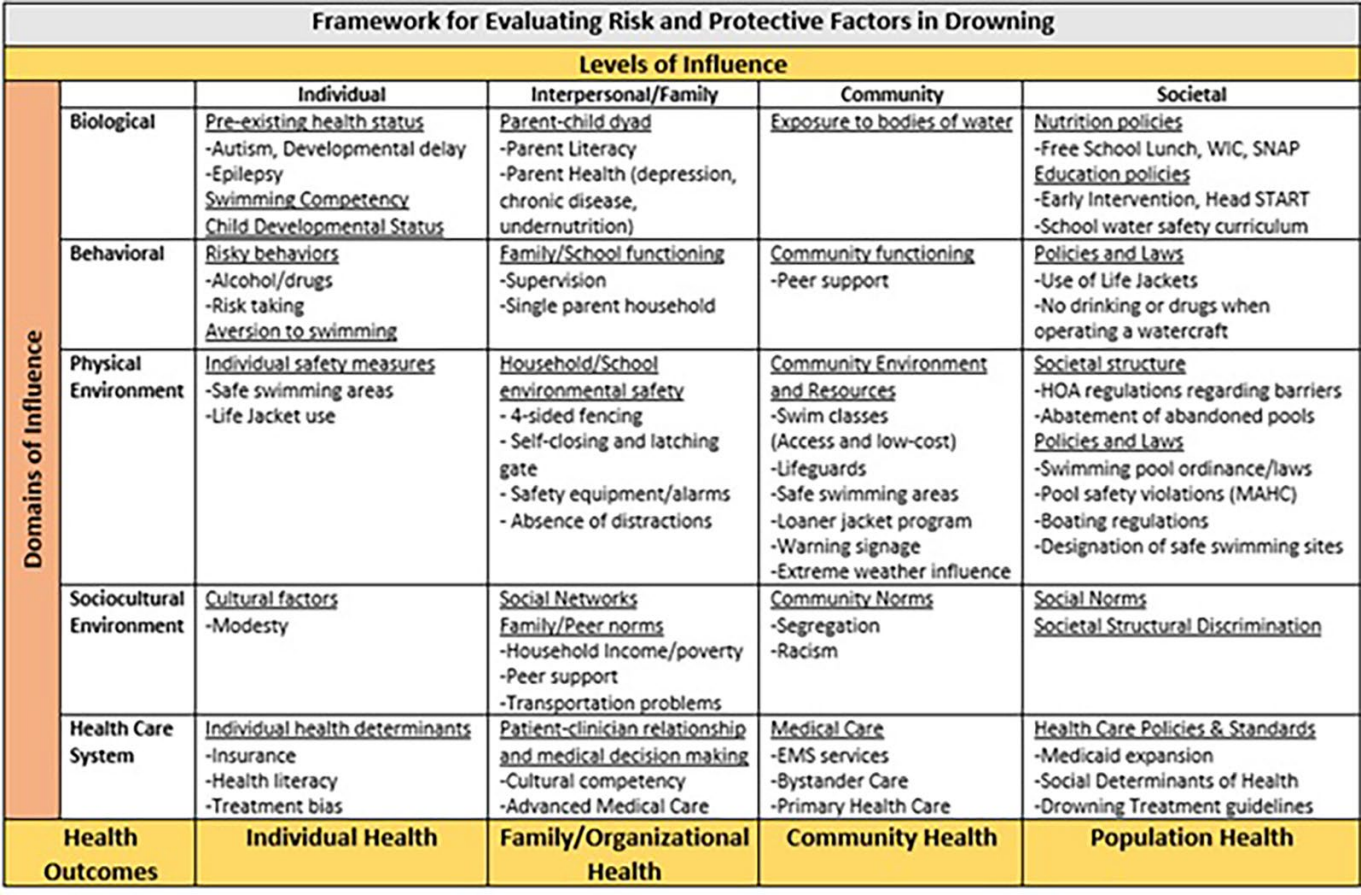
Framework for evaluating risk and protective factors in drowning

**Fig. 2:**
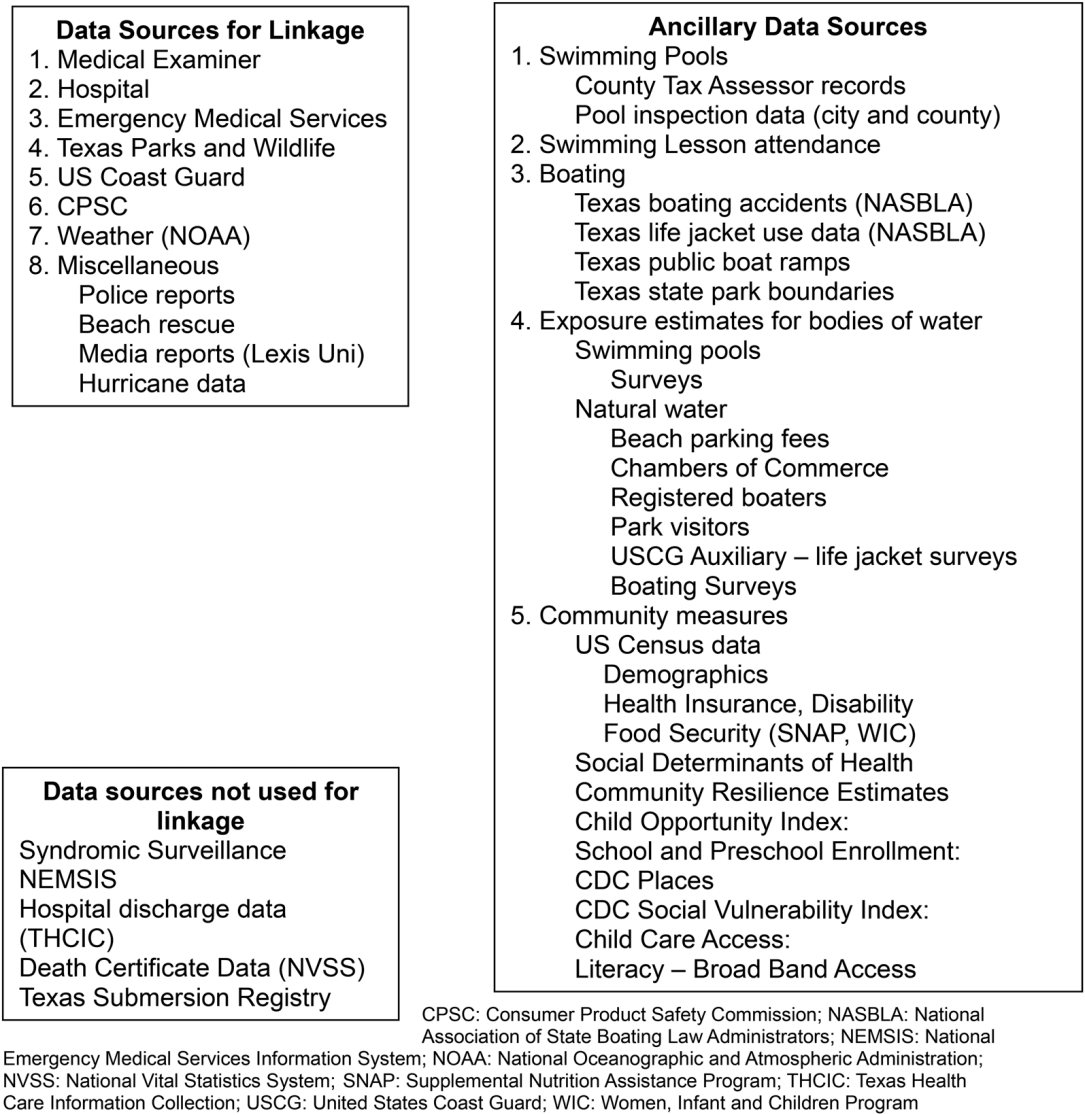
Data sources used to study fatal drowning in metropolitan Houston

The manner in which determinants of health influence drowning risk is not well understood [13]. To guide this investigation, we applied a framework that integrates the behavioral, physical, and socio-cultural factors in the population of interest and a modified version of the Haddon’s injury matrix (Fig. 1) [14, 15]. To accurately describe the epidemiology of drowning at local and regional levels, information on drowning events across all bodies of water, among both urban and rural populations, and across the continuum of care were captured (Fig. 2). Data used in this study consisted of EMS, hospital and medical examiner reports enriched with data from boating and maritime accidents, weather events, police and media reports, hurricane reports, beach rescue data, consumer-product safety reports, swimming pool inspections, census and land-use data, and health equity information.

## Data ownership and governance

The existing data sources were reviewed for data ownership and governance. This is important since the logistics, administrative issues and legal aspects involved with each dataset had to be anticipated to determine the timeline and costs associated with the overall project.

Data were obtained from multiple sources through freedom of information requests (US Coast Guard (USCG), Texas Parks and Wildlife (TPWL) [16], police reports), publicly available sources (Consumer Product Safety Commission (CPSC), National Oceanic and Atmospheric Administration (NOAA), county tax records, media reports, hurricane data, pool information from schools, municipalities, and non-profit organizations, and online searches) and data-use agreements (hospital, EMS and (medical examiner) ME records, syndromic surveillance data) for the Houston metropolitan region (Supplemental File 1). Police reports, hurricane and beach rescue data were merged with media reports.

*Open records/public information requests*: The Texas Public Information Act [17] grants the public the right to access governmental information. However, the governmental body is not required to answer factual questions, conduct legal research, or create new information. Applying for data is streamlined and can be done online. The governmental organization holding the data has to notify the requestor within 10 days of the request whether the data are available. Some jurisdictions seek the County Attorney’s determination before they can release records responsive to the request. This can delay data release. Justices of the Peace (JP), who conduct inquests for the smaller counties that lack medical examiners, are a part of the judiciary and are not subject to the Texas Public Information Act regarding release of open records data. The governmental organization provides an estimate of the charges that the requestor will incur if data are available. If data are readily available, data are usually supplied at no cost. Some entities, such as the USCG and TPWL utilize the Boating Accident Reporting Database (BARD) [18] have stipulations against data sharing. Inter-agency agreements at the Federal level may waive costs for Federally-funded research.

The Act allows the agency to furnish redacted data. In some cases, requests for data may yield no responsive information because (1) the database lacks an indicator, classification, or nature code for drowning; or (2) keyword searches using terms such as “drowning”, “death investigation”, or related terms return large volumes of irrelevant results.

Texas is a home rule state. Jurisdictional authority varies at the local, regional, and state levels. While each jurisdiction determines its own policies and regulations, some regulations, pertaining to life jacket use (Texas Parks and Wildlife) [19], swimming pool fencing (Texas Administrative Code [20]), and pool-related safety equipment (i.e., Virginia Graeme Baker Act-compliant drains) [21] are applicable statewide or nationally.

*Timeliness and availability of data*: Since this study was performed in a large metropolitan region and each of the eight counties had different administrative agencies tasked with providing information on drowning fatalities, EMS transports, hospital care, media, and police reports, it was impractical to obtain ethics approval and data use agreements with all respective agencies in the region. Hospital level data were obtained on patients who were treated in hospitals located in the larger counties (Harris County, Montgomery, and Fort Bend). Data on some patients who drowned in the smaller, more rural counties and who were transferred to medical facilities in the larger counties were also obtained during chart review. Supplemental File 1 describes the data availability by source.

*Classification of Body of Water*: The project aimed to identify risk groups by aquatic setting. Therefore, the bodies of water were classified to assess exposure among at-risk populations. The Houston metropolitan area encompasses diverse aquatic environments, such as recreational bodies of water, flood control structures, and residential settings (swimming pools, hot tubs, and bathtubs). The risk of drowning varies by body of water and depends on the number of persons in the immediate vicinity (i.e., those exposed) and the activity of the person. The following classification for bodies of water was used: swimming pool, hot tub, bathtub, natural water (beach, bay, lake, river), flood control structures (bayou, canal, creek, ditch, concrete waterway, reservoir, pond, retention pond), flooding event-related, and ‘Other.’ Boat-related and vehicle-related drowning were investigated separately within the body of water where the drowning occurred.

*Aquatic Exposure Information*: Exposure information was collected separately for constructed bodies of water (swimming pools, hot tubs, bathtubs) and natural water recreational sites (beaches, bays, lakes, rivers, and Galveston Bay).

Information on the type and location of swimming pools in the region were obtained electronically from tax records at the eight county tax appraisal districts, through written requests to the 46 school districts in the region, and by internet searches. Year 2020 US census data (at the block or block group level) were used when assessing population risk estimates. Swimming pool inspection data were only obtained for the largest county (i.e., Harris County) from the City of Houston [22] and the Harris County [23] Water Safety Departments.

Data on boating accidents were obtained from BARD maintained by the National Association of Boating Law Administrators (NASBLA) [24]. The locations of public boat ramps and state park boundaries were obtained from the Texas Land Office [25]. Information on boat marinas were obtained from Internet searches. The number of registered boaters by county and visitors at water recreational sites were obtained from the Texas Parks and Wildlife [16] and City of Houston Parks and Recreation Department [26]. Life jacket use and citizen boating exposure data were obtained from the U.S Coast Guard Auxiliary [27] which conducts annual life jacket use surveys at four popular natural water sites in the metropolitan Houston region (Lake Conroe, Galveston Bay, Clear Lake, and the San Jacinto river). From the survey dates, the number of motorized and human-powered watercraft were obtained and the annual number of boaters at these sites were estimated. The number of beach users was calculated using information from beach user parking fees, or by requests to the local Chambers of Commerce. Finally, community level data (Social Vulnerability Index (SVI) [28], Child Opportunity Index [29], census data) were obtained from online resources.

*Identification of Drowning Location*: Identifying the location of drownings was important for providing context to the events as well as providing information for local government and organizations involved in water safety. Drownings were geocoded to provide spatial information on the location of drowning.

Geocoding of drownings in which an address was provided (pools, bathtubs, hot tubs), geocoding was performed in two steps: (1) Address-matching to provide an approximate location; and (2) Matching the address to parcel information for which permanent pools had been identified. For drowning records without an address, especially those in natural water, the narrative was used to select the best approximation to the location. Some records (e.g., USCG) provided geographical coordinates for the drowning. For other records which provided the nearest street location for beach drownings, an estimate of where on the water’s edge the drowning occurred was made.

Exposure in recreational natural waters such as the beaches along the Gulf of Mexico coastline, lakes, rivers, smaller bays, and the large Galveston Bay was studied using the incident narratives to identify approximate location of drownings.

*Drowning risk and protective factors*: Information on drowning risk and protective factors were obtained from two sources: pre-existing data columns that were titled as such (e.g., swimming ability, life jacket use) or from the drowning circumstances present in the narratives of each drowning event among the datasets.

Hospital-level information on the drowning circumstances and risk and protective factors were obtained through medical chart review. This involved preparing, pilot testing and rolling out a standardized electronic clinical research form (eCRF) (Supplemental File 2) and training research staff in data abstraction. Research associates completed training and performed reliability testing and dual data entry to assure data quality. They reviewed medical records and entered relevant data into an eCRF.

The classification and attribute selection for risk and protective factors were based on existing literature. The attributes for supervision and barrier integrity were based on Anderson et al. [30] and Miller et al. [31] respectively. Other factors, such as life jacket use, CPR administration, past history of alcohol/drug use, drug/alcohol as a contributing factor, and comorbidities were assessed using the death scene investigative framework as described by Armstrong et al. [32]

*Missing Information*: The degree of missing data was assessed within each dataset and under-ascertainment of cases was assessed within the region. The Lincoln-Peterson estimator was used to measure the level of under-ascertainment among linked datasets.

## Technical environment

This project was performed in a university hospital. The hospital uses electronic medical records with capability to extract the data into a relational database. All systems are backed up daily so that databases could always be retrieved in case of events that caused loss or corruption of data files.

*Process documentation*: The data owner, data use agreement, data variable list, and method of data transfer (electronic or regular mail) was documented.

## Team skills and expertise

The project was directed by the principal investigator who was knowledgeable in drowning epidemiology, data sources, regulatory issues, and overall project management. Other team members included a social scientist specializing in spatial analysis (for spatial validation of drowning locations and determining the risk of drowning risk in subpopulations), a data scientist (to perform probabilistic linkage analysis), a statistician to perform conventional analysis, and a health economist to estimate the economic burden of drownings. Additional members included trained research associates who extracted data from medial charts and two site investigators, who supervised data abstraction at each of the two sites. A research director oversaw the regulatory aspects of the study and completion of data use agreements between cooperating agencies.

### Costs associated with obtaining data and data analysis

The costs associated with the project included administrative costs, computing platform and software, research staff effort, and the costs of data acquisition. The administrative costs, computer costs, and research staff effort were paid by the grant.

## (B) project execution

Project execution involved establishing data partnerships and data use agreements with relevant agencies and probabilistic linkage of data sets to provide a single, unified dataset for analysis. The methodology for probabilistic linkage is discussed elsewhere [10].

## Regulatory issues and ethics approval

It was necessary to ensure that safety standards, legal documents and processes pertaining to data use agreements and ethics review were maintained. IRB approval was necessary for obtaining patient-related information. This was sought and obtained from the three participating hospital systems and the Department of State Health Services (DSHS) Texas [33]. Consent was waived owing to the retrospective nature of the study. Data use agreements between participating entities covered data sharing, data storage, and data destruction practices. A deidentified dataset will be made available for research purposes only, subject to an explicit data sharing agreement. The files will be stored in a secure password protected web portal.

### Data security

Patient privacy and data security was a concern when linking datasets that had personal identifying and health information. This is especially true when performing deterministic linkage that uses personal identifying information [12]. In this study, the investigators utilized probabilistic linkage to ascertain drowning fatalities in the region because of the absence of personal identifiers in some datasets.

Linked cases were assigned a unique number and personal identifiers (e.g., name, date of birth, home address) were stripped to prepare a de-identified dataset for analysis. Database files were transferred electronically using file encryption such as secure file transfer programs (sFTPs) between research staff. Results were reported by sex, age group, and race/ethnicity and in aggregate when necessary to avoid re-identification of cases. Cell counts under ten were suppressed to prevent the identification of patients.

Data use agreements ensured that there were existing safeguards in the type and mode of data that was collected and transmitted. The investigators utilized web-based data entry and stored the data in a secure server with access limited to the research staff.

### Artificial intelligence and large Language modeling

Text parsing and OpenAI’s Large Language Model (LLM) were utilized to supplement unknown and missing data fields of risk and protective factors in data columns. The outputs of Open AI’s LLM were validated by manually analyzing the narratives for ground truth in approximately 20% of media report charts. Artificial intelligence testing was performed in a closed environment. OpenAI API, 2024, GPT-4o, date of use: May, 2025 was used. Studying the epidemiology of fatal drowning from media reports using artificial intelligence was not considered human subjects research by the Baylor College of Medicine IRB (H-56709).

### Data linkage

Probabilistic linking was performed using Python 3 and open-source libraries for comparison of string variables and to match records. Linkage between datasets began sequentially with datasets with most replete data followed by datasets with less complete data [10]. 

### Production and dissemination of products

The project developed a database of all permanent swimming pools in the eight-county region at the end of 2022. This was categorized by type of pool (i.e., single-family, multi-residential, school/college, recreational vehicle park, hotel/motel, community) and mapped to the corresponding land parcel. Drownings in natural waters were analyzed to identify problematic locations where there were concentrations of drownings. Research outputs, such as peer-reviewed publications, webinars, and seminars were produced.

## Results

Twelve datasets were reviewed; eight were successfully linked. Between 2016 and 2022 there were 790 drowning fatalities in metropolitan Houston. The median age was 40 years (IQR: 18.5,60); 71% were males. Children aged 0–17 years constituted 24% of drowning fatalities. Drownings occurred in swimming pools (27%), natural water (27%), flood control structures (20%), bathtubs (19%), and during flooding events (6%) [10]. 

### (A) project planning

#### Feasibility assessment

Data were available from EMS, hospital, and medical examiner report, boating and maritime accidents, weather events, police and media reports, hurricane reports, beach rescue data, consumer-product safety reports, swimming pool inspections, census and land-use data, and health equity information. Probabilistic linkage of multiple datasets produced an enriched analysis database which provided information on drowning events across all bodies of water, among both urban and rural populations, and across the continuum of care. Complete system-wide information on swimming class attendance, hospital encounters, and EMS transports and community-level regulations were unavailable given the numerous organizations that conduct these services in the entire eight-county region.

Data from the National EMS Information System (NEMSIS) reports [34], syndromic surveillance reports [35] and Texas Submersion Registry [36] could not be used for linkage with other datasets because records in these datasets were deidentified. Texas submersion registry data were provided in aggregate format and had a high degree of missing data that further limited its utility in the linkage project. Hospital discharge data could not be used for data linkage since regulations prohibit their linkage with other datasets.

Some data, such as the deceased individual’s beliefs, practices, family or household structure, swimming pool ownership, cultural norms (Fig. 1) were not analyzed since data were unavailable due to the retrospective nature of the study.

### Data ownership and governance

Existing data sources were reviewed for data ownership and governance to ascertain the logistics, administrative issues and legal aspects involved with each dataset. Table 1 describes the various data sources that were utilized in the study with their strengths and limitations and suggested methods to address their limitations. The smaller counties of Brazoria, Chambers, Liberty, and Waller, lack medical examiners. Death inquests in these counties are performed by the Justices of the Peace (JP). Requests to the JPs of these counties for drowning fatality information were not acknowledged. Therefore, police and media reports were obtained in these situations.

**Table 1:** Comparison of data sources used to study fatal drowning - Strengths, limitations and solutions

Datasets on Drowning			
Dataset	Strengths	Limitations/Technical/Legal/Data issues	Suggested Solutions
**Medical Examiner Fatality Data**	- Drowning information includes date/time of drowning and death, location, demographics, circumstances including risk and protective factors, environment -No cost -Not HIPAA governed	-Lag in obtaining final fatality reports -Confidentiality -Data formats differ between medical examiners -Missing data for protective and risk factors -Larger counties have medical examiners. Small counties have Justices of the Peace (JPs) where staff constraints limit data availability. -May need to purchase data	-Compare results with death certificate data (NVSS, and Texas DSHS) -Ensure data security -Obtain IRB and DUA -Standardize data format -Texas DSHS maintains a website with up to date information on fatal pediatric drownings by county, year, and sex
**Fatality data (Counties** **lacking a medical examiner)**	-No cost	-Small counties lack a medical examiner’s office. Justices of the Peace (JPs) conduct inquests; Funding and workforce constraints limit data availability. JPs are a part of the judiciary and the release of data is not subject to the Texas Public Information Act -Need to request each JP individually for data	-Request redacted information -Request data from police and media reports
**DSHS Texas Death Certificate Data**	-Accurate number of fatal drownings by demographics, county, ICD-10 codes, intent -Free online application	-Lengthy application process -Data available after DSHS Texas IRB approval -Onerous DUA -Data suppression for cell counts < 10	-Apply early -Be specific which data variables requested -Companion IRB from sponsoring institution helpful -Request multi-year aggregate data by age groups to avoid DUA
**Emergency Medical Services (EMS)**	-Drowning information includes date/time of drowning, geographic location, demographics, resuscitation, narrative -No cost -Cases identified by clinical assessment	-Missing data on intent, drowning circumstances -Data formats differ between EMS systems -Walk-in patients not included (may be > 25%) -Human subjects protections, IRB needed -Confidentiality -DUA finalization causes delays in obtaining data - Under-ascertainment bias based on EMS participation -Data transmission by secure file transfer (sFTP)	-Link with hospital data for accuracy -Standardize data format -Track patient disposition by receiving hospital -Obtain IRB and DUA - Ensure data security
**Hospital chart review**	- Drowning information includes date/time of drowning, location, demographics, resuscitation, narrative, outcome -Hospitalized and ED cases -No cost	-Misses cases declared dead at the scene -Under-ascertainment bias based on hospital participation; duplicate cases due to interfacility transfer or repeat hospital visits -Information on risk and protective factors may be incomplete -Post-ED care may be unknown -Data format between hospitals may differ -IRB and DUA necessary -Data programmer’s time requires funding	-Verify aggregate counts (and percentages) with hospital discharge data -Track patient disposition, especially interfacility transfers, by reviewing narratives, transfer memos -Ensure data security -Standardize data format -Reliance IRB between institutions
**Texas Parks and Wildlife** **(TPWL)**	-No cost (available online) -Natural water drowning per Boating Accident Report Database (BARD) -Drowning date/time, county of drowning, circumstances (risk/protective factors, environment)	-Missing race, ethnicity, age	-Link with NOAA, fatality, and other databases -USCG and TPWL are similar data based on Boating Accident Report Database (BARD)
**US Coast Guard**	-Cost based on data request -Data available if classified as functional request” via FOIA. -Natural water drownings per Boating Accident Report Database (BARD) -USCG data more complete than TPWL data --Drowning date/time, county of drowning, circumstances (risk/protective factors), water and weather conditions, boat’s condition, cause of accident, number dead or injured	-Missing data on race, ethnicity, and swimming ability -Need to obtain functional request from Texas Parks and Wildlife Department (TPWD) to release records based upon requested fields - Database and records cannot be released per TPWD (Statistics from extract can be generated) and shared). -Data transmission by secure file transfer	-Link with NOAA, fatality, and other databases -USCG and TPWL are similar data based on Boating Accident Report Database (BARD)
**NOAA**	-No cost, available online -Weather conditions (floods, storms, rip currents) available by date/time, location (GIS), short narrative, data source -Number dead or injured	-Scant/no demographic information	-Link with other databases
**Media reports**,** Hurricane data**,** Beach rescue data**	-No cost, online/request -Drowning circumstances (risk/protective factors, circumstances), location (GIS), demographics -Outcomes (fatal/non-fatal) -Useful for smaller counties	-Cannot track all fatal cases -Under-reporting and biased toward natural water drowning or flooding events -Subjective/missing information on drowning risk and protective factors	-Link with other databases
**CPSC data**	-Free, available online -Provides information on month/year of drowning, ICD-10 code, product code, demographics, brief narratives, outcome (fatal/nonfatal), data source	-Does not provide date of injury -Only consumer products (constructed bodies of water i.e., pools, bathtubs, etc.)	-Link with other databases
**Pool Violation Data (City and County)**	-No cost	-Variable pool inspections and reporting by cities and counties -Does not provide information about above-ground or portable pools	-Verify with medical examiner reports -Verify NEISS data for portable pool violations using product codes (3221, 3251 and 5043)
**Pool Location Data (County Tax Appraisal Data)**	-No cost -Classifies swimming pools based on use (private residential or commercial (apartment properties)	-Does not provide information for portable pools -Some entities are tax exempt (school districts, non-profits) and do not appear in appraisal district data	-Verify NEISS data for portable pool violations using product codes (3221, 3251 and 5043) -Use Google Earth to locate pools -File FOI request to the Texas Education Agency (TEA) for locations of all private and public schools in the region with pools or natatoria
**Law Enforcement call logs (Police and Sheriff’s Departments)**	-No cost -Provides information on date/time of drowning, geographic location, intent, body of water, demographics, and outcomes (fatal/non-fatal)	-Lack specific information of drowning victim -Missing data common	-Request redacted information
**Hospital and ED/Outpatient Discharge Data (Administrative Data - THCIC)**	-Provides aggregate data on demographics, age groups, ICD-10 and CPT codes, hospital charges, outcomes (fatal and non-fatal) -Hospital and stand-alone EDs -Data for purchase -All drowning cases by county of treatment	-Lag in obtaining data (1 year) -Linking to personal identifiers prohibited -No information on drowning circumstances -Duplicate cases due to repeat encounters or interfacility transfers -Diagnoses, procedures, ethnicity, payer may not be standardized -Hospital charges different from billed charges -Expensive	-Ensure data security -Track admission and disposition and transfers from stand-alone EDs -Check inpatient dataset for mode of admission to determine if patient admitted from the ED. -Obtain IRB -Use cost-charge ratios to ascertain billed charges
**Syndromic Surveillance**	-Provides daily drowning burden and case demographics -Deidentified data -ED and urgent care visits for drowning -Cases based on ICD-10 codes, chief complaint, or discharge diagnosis	-Provides only visit date and case demographics -Unable to differentiate cases based on intent and outcome; duplicate cases may arise due to interfacility transfers or repeat hospital visits -Unable to link data with other datasets -IRB and DUA required -Completeness of data depends on degree of participation by all facilities in the region -Data confidentiality -Data transfer by secure file transfer (sFTP)	-Verify case burden and demographics with hospital discharge data -Verify fatal cases with medical examiner data -Ensure data security
**NEMSIS**	-No cost -Deidentified and aggregate data	-Unable to link data -Can perform home to incident analysis	-Exploratory
**Texas Submersion Registry**	-No cost -Online request -Hospital and EMS data -Demographics, age group, body of water, outcome	-Incomplete and missing data due to -Incomplete participation in registry by entities -Suppression of data with cell counts < 5 -Cannot differentiate between EMS or hospital cases or based on outcome	-Verify with hospital discharge data

THCIC: Texas Health Care Information Collection, NEMSIS: National Emergency Medical Services Information System, NOAA: National Oceanographic and Atmospheric Administration, DSHS: Department of State Health Services, CPSC: Consumer Product Safety Commission

*Open records/public information requests*: The procedure to obtain relevant data was time-consuming and challenging given the disparate data sources for fatal and non-fatal drowning, water recreational bodies, enforcement agencies, and socio-demographic information of the at-risk population. Data were often unavailable, unstandardized, or missing key information. This was especially true for smaller counties that lacked financial resources and manpower. The larger counties generally maintain good record keeping practices and had resources to provide requested information electronically.

*Timeliness and availability of data*: Recognizing the challenge with obtaining complete data from multiple agencies and because this was an epidemiological study, the investigators decided to utilize five key variables (age, sex, date of drowning/death, county of drowning, and body of water) for performing probabilistic linkage of multiple datasets. These five variables, which are readily available, had the least proportion of missing information and allowed successful probabilistic linkage which, in turn, enabled the identification of some associated drowning risk and protective factors.

The investigators followed up on their freedom of information requests for fatal drowning data from the governmental authorities as needed. Some data requests (e.g., USCG) required routing through the proper channels and took several months to fulfill. In contrast, online sources, including TPWL, NOAA, CPSC, hurricane data and media reports were available either immediately or within a short period of time. Medical examiner, hospital, and EMS data for the larger counties were promptly available after IRB approval and completion of data use agreements.

*Aquatic Exposure Information*: Exposure information was available for constructed bodies of water and natural water recreational sites. Exposure data for flood-control structures such as retention ponds and bayous could not be estimated because there are no data sources with this information and it is difficult to estimate the number of persons who visit these sites.

Information on the type and location of swimming pools in the region were obtained electronically from tax records at the eight county tax appraisal districts. Swimming pool inspection data were only available for the largest county (i.e., Harris County) from the City of Houston [22] and the Harris County [23] Water Safety Departments.

Data on boating accidents, locations of public boat ramps and state park boundaries, boat marinas, number of registered boaters by county and visitors at water recreational sites, life jacket use and citizen boating exposure data, estimates of the number of motorized and human-powered watercraft, annual number of boaters at 4 popular natural water recreational sites, and beach users were easily obtained from the relevant authorities.

Finally, community level data were readily available from the US Census Bureau.

*Identification of drowning location*: Spatial identification of drowning locations was possible in 97% of drowning events [10]. Spatial accuracy of the location of drowning events where the address was provided was high. Errors tended to be fewer with those being mostly from transcription (e.g., incorrect address listed, misspellings).

For those records without an address, estimation error was greater than for drowning locations with addresses. Spatial geocoding enabled identification of drownings that occurred outside the region and to enhance the description of body of water where the drowning occurred [10]. 

Incident narratives which described the approximate location of drowning were used to study exposure in recreational natural waters such as the beaches along the Gulf of Mexico coastline, lakes, rivers, smaller bays, and the large Galveston Bay. Unfortunately, with these drownings, information was often lacking or poorly described. Errors included vague descriptions of locations, inconsistent terminology (e.g., mixing up beaches on the Gulf of Mexico with bay or confusing piers with jetties), no quantification of distance, and no descriptions of direction from a reference point. Only a few cases provided actual coordinates for the drowning. A lack of standardization in geographical information proved to be a problem.

*Drowning risk and protective factors*: Not all data sources provided information on the risk and protective factors and, if they did provide this information, often presented it in an unstandardized form. Some data sources like the Harris County Medical Examiner data provided both structured data and narratives of drowning circumstances. Medical examiner data from other counties were restricted to demographics with a brief description of the drowning event. EMS transport data provided a clinical impression and information on resuscitation, outcome, and a brief description of the drowning circumstances. Narratives were also available from CPSC, media reports, and NOAA reports.

Hospital records provided varying degree of information on drowning risk factors. Information on supervision, resuscitation, and comorbidities was more frequently documented compared to documentation of swimming ability, life jacket use, presence of barriers, and concurrent alcohol or drug use. Toxicology reports corroborated drug/alcohol use.

*Missing Information*: Missing information was a significant concern. This was due to missing data within each dataset and under-ascertainment of cases within the region, mostly because of the project’s inability to obtain and combine data from all relevant agencies. There was a low proportion of missing data for the five variables (i.e., age, sex, county of drowning, date of drowning and body of water) that were used for probabilistic linkage [10]. However, the degree of missing data for the drowning risk and protective factors was higher, even after probabilistic linkage [10]. 

Because of the degree of missing information, it was necessary to report on the total error which includes both uncertainty from missing information as well as uncertainty due to sampling error [37]. Both have to be considered when estimating the extent to which risk and protective factors affect drowning outcomes. The under-ascertainment rate of fatal drowning by individual dataset varied from 0.02 to 0.46 based on the Lincoln-Petersen estimator.

### Costs associated with obtaining data and data analysis

Data were obtained free of charge from publicly available data sources (i.e., TPWL, NOAA, hurricane data, media reports, CPSC). There were minimal costs associated with obtaining data through freedom of information requests, which was related to the amount of time necessary by office personnel to access and retrieve data (physically or electronically). Data from one hospital system were purchased for $1,000. Research staff effort for probabilistic linkage and spatial analysis were paid by the grant.

### Regulatory issues and ethics approval

One issue faced by the research team was the lengthy application process to obtain DSHS Texas IRB approval for drowning-related death certificate data for the region of interest. The application took almost two years to be approved. After IRB approval, DSHS Texas stipulated that individual case data could only be released after a rigorous data sharing agreement was completed between DSHS and the investigator’s institution. Per this non-negotiable data use agreement, the principal investigator would have been responsible for reporting any data breaches immediately upon discovery of the breach. Given these exacting requirements, the investigators opted to abandon their request for individual case data and instead requested deidentified, aggregate data from DSHS Texas. These data were used to compare injury burden and demographics between DSHS Texas data and probabilistically linked data.

### Dissemination of products

The project developed or is developing several products. First, a database of all permanent swimming pools in the eight-county region at the end of 2022 categorized by type of pool (i.e., single-family, multi-residential, school/college, recreational vehicle park, hotel/motel, community) and mapped to the corresponding land parcel was shared with the City of Houston Water Safety and Harris County Public Health departments. This database, which provides the geographical coordinates of parcel with permanent swimming pools, can assist public health authorities in performing spatial analysis of problematic pools such as those that have passed or failed safety inspections. Second, drownings in natural waters were analyzed to identify problematic locations with concentrations of drownings. Separate analyses are being done on these locations to identify probable risk factors and the analysis will be provided to local governments and organizations involved in water safety. Third, research outputs, such as peer-reviewed publications, maps, webinars, and seminars are/will be uploaded on a webpage and shared with the research community and the public. (Project ASTRAL https://www.bcm.edu/departments/pediatrics/divisions-and-centers/emergency-medicine/research)

## Discussion

This paper documents the approaches in obtaining data from multiple sources, the strengths and limitations of each dataset, and the preparatory steps before conducting probabilistic data linkage to describe the epidemiology of fatal drowning in a large metropolitan region. The methodology can be replicated to study other injuries.

A reason the epidemiology of drowning in the United States has been understudied is because there is no current single data source which provides complete data on fatal and non-fatal drowning. This makes it difficult to describe contextual information of drowning incidents which is necessary when developing drowning countermeasures. The USNWSAP recommends linking drowning data across the drowning spectrum [38]. Probabilistic linkage of multiple datasets is a possible solution.

When performing data linkage, it is important to first perform a comprehensive search of all possible data sources that represent all bodies of water, urban and rural communities, and drowning incidents across the continuum of care (i.e., EMS, hospital, and medical examiner). Probabilistic linkage combines data from several sources. The linkage allows each dataset to complement data that is missing in other datasets and to identify new and unique injury cases. This process reduces ascertainment bias and produces an enriched analysis database.

While probabilistic data linkage is possible with fatal drowning, it may be more challenging to utilize this methodology to study the epidemiology of non-fatal drowning. Within a region, patients of non-fatal drowning may access a myriad of healthcare agencies, such as EMS, hospitals, and clinics, each of which have their unique data system and regulatory requirements. This makes it difficult for the researcher to enter into multiple data sharing agreements. Procuring data is time consuming. Therefore, data requests should begin early in the research project to allow sufficient time to obtain ethical and institutional approval and to negotiate data use agreements with the relevant agencies. Drowning is a relatively rare event. Therefore, several years of data are necessary when studying injury burden and the groups at risk for drowning.

Data cleaning and ensuring that data variables are standardized across datasets is essential prior to performing probabilistic linkage. This is a time-intensive process. An important contributor for unstandardized data is a lack of consensus on a common list of variables to be used for studying drowning epidemiology. Widespread use of data variables and associated terminology present in the Utstein classification and the Child Death Review form [7, 39] could help in standardizing data elements.

Once data procurement, data cleaning and data standardization are completed, probabilistic linkage of datasets can commence. Data linkage should begin with datasets that contain the most complete information (e.g., medical examiner reports, death scene investigations, EMS, and hospital records). The location of drowning and the body of water involved should be identified to allow protective measures to be developed. These data can be used for hot spot analysis and the identification of high risk drowning locations. Artificial intelligence (large language models) may be helpful in extracting information on drowning risk and protective factors from the narratives present in medical examiner’s reports.

An existing limitation of drowning epidemiology is that the risk of drowning is expressed at the population level rather than being exposure-based. Since the distribution of swimming pools or access to natural water recreational areas is very uneven in most metropolitan areas, using population as a baseline for within-region comparisons can be misleading. In this study, drowning risk was ascertained from available water-related data such as the number of swimming pools, estimated boaters, or beach users. Data for these measures have to be obtained from a plethora of data sources. While not perfectly describing actual exposure, they provide a better framework for evaluating drowning risk than using population by itself. Process documentation is crucial so that future researchers can benefit from completed research projects.

Finally, researchers must fulfil statutory requirements for obtaining, reporting, and sharing data. Ethics approval for conducting research and the completion of data use agreements between the agencies providing the data and researchers is required, especially if the data involves personal protected information. To safeguard against identification of patients, the reporting of results have to be suppressed when the injury counts are low.

This paper has important public implications. First, it documents the data sources, processes, and challenges that need to be addressed in order to conduct successful data linkage when studying fatal drowning. This can benefit public health professionals and researchers who wish to study the epidemiology of injuries and to conduct injury countermeasures. Second, data on drowning are often disparate, incomplete, and unstandardized across datasets and it is necessary to utilize uniform terminology. Data variables need to be standardized across the drowning continuum of care. There is a need for widespread use of the Utstein template [39] and the child death reporting form [7], by the offices of the medical examiner. Increased emphasis on standardization of geographical information across agencies could improve the spatial accuracy of natural water drowning locations.

Third, in order to obtain timely data, data should be stored electronically and have uniform search terms that can assist chart retrieval. Fourth, data on natural water drowning that are stored in BARD (TPWL and USCG) lack race and ethnicity information of persons who drown. Collection of information on race and ethnicity in BARD may help identify high risk groups who drown in natural water. Fifth, tracking patients across the continuum of care is challenging and could be improved by implementing a uniform identification number. Finally, probabilistic linkage, spatial analysis and exposure-adjusted risk determination could be utilized by other researchers who intend to investigate other types of injuries.

The study has limitations. First, the study was performed in a single region of the United States where the waterscapes, administrative agencies and regulations may differ compared to other regions in the country. However, the approaches for data acquisition and analysis are likely generalizable. Second, the study was performed as a research project. In order for probabilistic linkage to be expanded to the regional or state level and assist in drowning surveillance, it would be necessary to improve current analytic tools, expand the capacity of local and state agencies in data science, provide funding, address workforce constraints, and improve the timely dissemination of linked data [40]. Third, missing data was a concern, especially data pertaining to the risk and protective factors. Furthermore, we were unable to obtain data from unconventional data sources such as social media. However, verifying the authenticity of this type of information is challenging. Fourth, we could not report on individual’s beliefs, practices, family or household structure, cultural norms, and community-level regulations across the region. While these factors have a bearing on drowning outcomes, the relative contribution of these factors on outcomes was beyond the scope of this project and will need further study. Finally, nonfatal drownings were not studied in this project.

### Future directions

This linkage project is an initial step in identifying the barriers and solutions for performing drowning surveillance at the regional level. The next steps would be to scale linkage methodology by increasing the availability of timely data, standardizing drowning variables, documenting and improving analytic tools and processes, and expanding the capacity of state and regional agencies in data science methodologies [38, 40]. Additionally, research outputs will be disseminated to public health authorities, academia, and the general public.

### Conclusions

There are a plethora of data sources for fatal drowning. The process of obtaining and analyzing data to describe the epidemiology of fatal drowning using probabilistic data linkage is complex, lengthy, and cumbersome. Documenting the process and lessons learned can support drowning research and inform regional drowning prevention strategies.

## Supplementary Information

Supplementary Material 1.

Supplementary Material 2.

## Data Availability

Data will be available upon conclusion of the grant and study period on October 1, 2026 and upon request and data use agreement.
