# Supplementary material for: Lessons learned in a data linkage project on fatal drowning
Source: Inj Epidemiol. 2026 Feb 1;13:17. doi: 10.1186/s40621-026-00660-x (PMC12951990; doi:10.1186/s40621-026-00660-x)
Supplement: Supplementary file 1 — Supplementary Material 1. [file 40621_2026_660_MOESM1_ESM.pdf]

Supplemental File 1: Drowning Data Sources

Data Source, Procurement	Body of Water	Data Source
1. Medical Examiner (IRB)	All	Counties: Harris, Fort Bend, Galveston, Montgomery
2. Hospital (IRB and DUA)	All	Three hospital systems
3. EMS (IRB and DUA)	All	Houston EMS, Three Harris County EMS agencies
4. Police Reports (FOI)	All	Sheriff's office (Galveston County, Chambers County, Brazoria County, Fort Bend County) and Houston Police Department
5. Texas Parks and Wildlife (FOI)	Natural water, water-craft, water-transport	https://tpwdtexas.govqa.us/WEBAPP/_rs/(S(nnqh5hgo3rdqhmpmtym1ydsz))/SupportHome.aspx
6. Maritime (USCG) (FOI)	Natural water, water-craft, water-transport	US Coast Guard
7. Weather (NOAA) (Publicly available)	Weather-related, rip-current-related, flood	https://www.ncdc.noaa.gov/stormevents/
8. Beach Rescue (Publicly available)	Rip current, Gulf	Galveston Beach Patrol
9. Media Reports (Publicly available)	All	Multiple internet sites, Lexis Uni, Houston Chronicle
10. CPSC (Publicly available)	Swimming pool, hot tub	Clearinghouse@cpsc.gov
11. Hurricane data (Publicly available)	Flood-related drowning.	https://data.4tu.nl/articles/Fatalities_due_to_hurricane_Harvey_2017_/12708032
12. NVSS (Publicly available)	All	CDC Wonder: https://wonder.cdc.gov/
13.Syndromic Surveillance (IRB and DUA)	All	Houston Health Department
14. NEMSIS (Publicly available)	All	https://nemsis.org/

County<br>Data Source	Brazoria	Chambers	Fort Bend	Galveston	Harris	Liberty	Montgomery	Waller
Medical Examiner	No	No	Yes*	Yes	Yes	No	Yes*	No
Hospital	Yes	Yes	Yes	Yes	Yes	Yes	Yes	Yes
EMS	No	No	No	No	Yes	No	No	No
Media	Yes	Yes	Yes	Yes	Yes	Yes	Yes	Yes
Police Reports	Yes	Yes	Yes	Yes	Yes	No	No	No
Texas Parks	Yes	Yes	Yes	Yes	Yes	Yes	Yes	Yes
US Coast Guard	Yes	Yes	Yes	Yes	Yes	Yes	Yes	Yes
CPSC	Yes	Yes	Yes	Yes	Yes	Yes	Yes	Yes
NOAA	Yes	Yes	Yes	Yes	Yes	Yes	Yes	Yes
Syndromic Surveillance	Yes	Yes	Yes	Yes	Yes	Yes	Yes	Yes

DUA: Data use agreement; FOI: Freedom of Information request; CPSC: Consumer Product Safety Commission; NOAA: National Oceanographic and Atmospheric Administration; EMS: Emergency Medical Services; NVSS: National Vital Statistic System; NEMSIS: National Emergency Medical Services Information System.
*some years
