# Supplementary material for: Lessons learned in a data linkage project on fatal drowning
Source: Inj Epidemiol. 2026 Feb 1;13:17. doi: 10.1186/s40621-026-00660-x (PMC12951990; doi:10.1186/s40621-026-00660-x)
Supplement: Supplementary file 2 — Supplementary Material 2. [file 40621_2026_660_MOESM2_ESM.pdf]

Data Dictionary Codebook

ASTRAL_version1 (PID: 13088)

07/31/2025 8:25am

**Instruments**	
Instrument	Form Name
Demographics and Submersion details	demographics_and_submersion_details

	#	Variable / Field Name	Field Label<br>*Field Note*	Field Attributes (Field Type, Validation, Choices, Calculations, etc.)
**Instrument: Demographics and Submersion details (demographics_and_submersion_details)**				
	1	[study_id]	Study ID	text
	2	[abstractor]	Data abstractor last name	text, Required<br>Custom alignment: LV
	3	[pref_lang]	Patient's Preferred Language (Language spoken at home)	radio<br>1 English<br>2 Spanish<br>3 Vietnamese<br>4 Chinese<br>5 Hindi, Urdu or Bengali<br>6 Arabic<br>7 French<br>66 Other<br>88 Unknown<br>Custom alignment: LH
	4	[other_lang]<br>Show the field ONLY if:<br>[pref_lang] = '66'	If Other, specify Language	text<br>Custom alignment: LH
	5	[urgent_care]	Was the patient seen in URGENT CARE or MEDICAL CLINIC? (If YES, DO NOT complete the questions about Hospital-related information and go directly to complete the demographic and drowning details)	radio, Required<br>1 Yes<br>0 No<br>88 Unknown<br>Custom alignment: LH
	6	[ems_call]<br>Show the field ONLY if:	Was EMS summoned to the scene?	radio, Required<br>1 Yes<br>0 No

		[urgent_care] = '0' or<br>[urgent_care] = '88'		88 Unknown<br><br>Custom alignment: LH
	7	[scene_transport]<br><br>Show the field ONLY if:<br>[urgent_care] = '0' or<br>[urgent_care] = '88'	Was the patient transported by EMS to the First Hospital?	radio, Required<br>1 Yes<br>0 No<br>99 Unknown<br><br>Custom alignment: LV
	8	[non_transportems]<br><br>Show the field ONLY if:<br>[scene_transport] = '0'	If Non-transport by EMS to a hospital, what was the disposition of the victim from the scene?	radio, Required<br>1 Healthy and discharged from scene<br>2 Dead at scene and transported by coroner to morgue<br>3 Transported by private auto to the Clinic, ER or Hospital<br>88 Unknown<br><br>Custom alignment: LV
	9	[mode_arrival_first_hosped]<br><br>Show the field ONLY if:<br>[urgent_care] = '88' or<br>[urgent_care] = '0'	What was the mode of arrival to the FIRST hospital ED?	radio, Required<br>1 EMS, Life Flight<br>2 Walk-in, Private Auto<br>88 Unknown<br><br>Custom alignment: LV
	10	[ems_date]<br><br>Show the field ONLY if:<br>[mode_arrival_first_hosped]=1	What was the date of EMS transport?	text (date_mdy, Min: 2016-01-01, Max: 2023-12-31), Required<br>Custom alignment: LH<br>Field Annotation: @HIDEBUTTON
	11	[hospital1]<br><br>Show the field ONLY if:<br>[urgent_care] = '0' or<br>[urgent_care] = '88'	Presenting Hospital (1st Hospital)	dropdown, Required<br>10 Texas Children's Hospital<br>11 Texas Children's Hospital West Campus<br>12 Texas Children's Hospital Woodlands<br>13 Memorial Hermann TMC<br>14 Memorial Hermann Children's<br>15 Memorial Hermann Southwest<br>16 Memorial Hermann Northwest

				17 Memorial Hermann Memorial City<br>18 Memorial Hermann Katy<br>19 Memorial Hermann Sugarland<br>20 Memorial Hermann Heights<br>21 Memorial Hermann Woodlands<br>22 Ben Taub Hospital<br>23 LBJ Hospital<br>24 Clear Lake Regional HCA<br>25 Tomball Hospital HCA<br>26 Conroe Regional HCA<br>27 Kingwood HCA<br>28 East Houston HCA<br>29 Bayshore Hospital<br>30 St. Luke's TMC<br>31 St. Luke's Woodlands<br>32 St. Luke's Heights<br>33 St. Luke's Sugarland<br>34 St. Luke's Vintage<br>35 West Houston<br>36 Methodist TMC<br>37 Methodist West Houston<br>38 Methodist Baytown<br>39 Methodist Willowbrook<br>40 Methodist Katy<br>45 Methodist Woodlands<br>46 Brazosport Regional<br>47 Bryan College Station<br>48 St. Elizabeth Beaumont<br>49 Women's Hospital<br>50 St. John's Nassau Bay<br>67 Stand-alone Emergency Department (Neighbor's, Elite Care, etc.)<br>66 Other<br><br>Custom alignment: LV

	12	[hosp1_othr]<br><br>Show the field ONLY if:<br>[hospital1] = '66'	Other presenting hospital (1st Hospital)	text, Required<br>Custom alignment: LV
	13	[dispo_hosp1]<br><br>Show the field ONLY if:<br>[urgent_care] = '0' or [urgent_care] = '88'	What was the disposition from Presenting hospital (1st Hospital)?	dropdown, Required<br>1 Seen in ED<br>2 Observed in Short-Stay (Observation Unit) at 1st hospital<br>3 Admitted in Acute Care ward in 1st hospital<br>4 Admitted to PICU in 1st hospital<br>5 Transferred to other facility/2nd hospital<br>6 AMA discharge (Discharge against medical advice)<br>7 Died<br>88 Unknown<br>66 Other<br><br>Custom alignment: LH
	14	[dispo_hosp1_othr]<br><br>Show the field ONLY if:<br>[dispo_hosp1] = '66'	Other disposition from presenting hospital (1st Hospital)	text, Required<br>Custom alignment: LH
	15	[receiv_hosp_2]<br><br>Show the field ONLY if:<br>[dispo_hosp1] = '5'	Receiving Hospital (2nd hospital)	dropdown, Required<br>10 Texas Children's Hospital<br>11 Texas Children's Hospital West Campus<br>12 Texas Children's Hospital Woodlands<br>13 Memorial Hermann TMC<br>14 Memorial Hermann Children's<br>15 Memorial Hermann Southwest<br>16 Memorial Hermann Northwest<br>17 Memorial Hermann Memorial City<br>18 Memorial Hermann Katy<br>19 Memorial Hermann Sugarland<br>20 Memorial Hermann Heights<br>21 Memorial Hermann Woodlands

				22 Ben Taub Hospital<br>23 LBJ Hospital<br>24 Clear Lake Regional HCA<br>25 Tomball Hospital HCA<br>26 Conroe Regional HCA<br>27 Kingwood HCA<br>28 East Houston HCA<br>29 Bayshore Hospital<br>30 St. Luke's TMC<br>31 St. Luke's Woodlands<br>32 St. Luke's Heights<br>33 St. Luke's Sugarland<br>34 St. Luke's Vintage<br>35 West Houston<br>36 Methodist TMC<br>37 Methodist West Houston<br>38 Methodist Baytown<br>39 Methodist Willowbrook<br>40 Methodist Katy<br>45 Methodist Woodlands<br>46 Brazosport Regional<br>47 Bryan College Station<br>48 St. Elizabeth Beaumont<br>49 Women's Hospital<br>50 St John's Nassau Bay<br>66 Other<br>67 Stand-alone Emergency Department<br><br>Custom alignment: LV
	16	[hosp2_othr]<br><br>Show the field ONLY if:<br>[receiv_hosp_2] = '66'	Other Receiving hospital (2nd Hospital)	text, Required<br>Custom alignment: LV
	17	[dispo_hosp2]<br><br>Show the field ONLY if:<br>[dispo_hosp1] = '5'	What was the disposition from the Receiving hospital (2nd Hospital)?	dropdown, Required<br>1 Seen in ED<br>2 Observed in Short-Stay (Observation Unit) at 2nd

				hospital<br>3 Admitted in Acute Care ward in 2nd hospital<br>4 Admitted to PICU in 2nd hospital<br>5 Transferred to other facility<br>6 AMA discharge (Discharge against medical advice)<br>7 Died<br>88 Unknown<br>66 Other<br><br>Custom alignment: LH
	18	[dispo_hosp2_othr]<br><br>Show the field ONLY if:<br>[receiv_hosp_2] = '66'	Other disposition from receiving hospital (2nd Hospital)	text, Required<br>Custom alignment: LH
	19	[receiv_hosp_3]<br><br>Show the field ONLY if:<br>[dispo_hosp2] = '5'	Receiving Hospital (3rd hospital)	dropdown, Required<br>10 Texas Children's Hospital<br>11 Texas Children's Hospital West Campus<br>12 Texas Children's Hospital Woodlands<br>13 Memorial Hermann TMC<br>14 Memorial Hermann Children's<br>15 Memorial Hermann Southwest<br>16 Memorial Hermann Northwest<br>17 Memorial Hermann Memorial City<br>18 Memorial Hermann Katy<br>19 Memorial Hermann Sugarland<br>20 Memorial Hermann Heights<br>21 Memorial Hermann Woodlands<br>22 Ben Taub Hospital<br>23 LBJ Hospital<br>24 Clear Lake Regional HCA<br>25 Tomball Hospital HCA<br>26 Conroe Regional HCA<br>27 Kingwood HCA<br>28 East Houston HCA

				29 Bayshore Hospital<br>30 St. Luke's TMC<br>31 St. Luke's Woodlands<br>32 St. Luke's Heights<br>33 St. Luke's Sugarland<br>34 St. Luke's Vintage<br>35 West Houston<br>36 Methodist TMC<br>37 Methodist West Houston<br>38 Methodist Baytown<br>39 Methodist Willowbrook<br>40 Methodist Katy<br>45 Methodist Woodlands<br>46 Brazosport Regional<br>47 Bryan College Station<br>48 St. Elizabeth Beaumont<br>49 Women's Hospital<br>50 St John's Nassau Bay<br>66 Other<br><br>Custom alignment: LV
	20	[hosp3_othr]<br><br>Show the field ONLY if:<br>[receiv_hosp_3] = '66'	Other Receiving hospital (3rd Hospital)	text, Required<br>Custom alignment: LV
	21	[dispo_final]<br><br>Show the field ONLY if:<br>[urgent_care] = '0' or [urgent_care] = '88'	Patient's final disposition from hospital	radio, Required<br>1 Home<br>2 Rehabilitation Facility or Hospice<br>3 Morgue<br>4 Discharge against medical advice<br>66 Other<br>88 Unknown<br><br>Custom alignment: LH
	22	[dispo_final_uc]<br><br>Show the field ONLY if:<br>[urgent_care] = '1'	Patient's disposition from urgent care	radio, Required<br>1 Home in good condition<br>2 ER<br>66 Other

				88 Unknown<br><br>Custom alignment: LH
	23	[dispo_final_uc_othr]<br><br>Show the field ONLY if:<br>[dispo_final_uc] = '66'	Specify other patient's disposition from urgent care	text, Required<br>Custom alignment: LV
	24	[dispo_final_othr]<br><br>Show the field ONLY if:<br>[dispo_final] = '66'	Specify other patient's final disposition	text, Required<br>Custom alignment: LV
	25	[date_death]<br><br>Show the field ONLY if:<br>[dispo_final] = '3' or [non_transportems]= 2	What was the date of Death?	text (date_mdy, Min: 2016-01-01, Max: 2023-12-31), Required<br>Custom alignment: LH<br>Field Annotation: @HIDEBUTTON
	26	[neuroldischstatus]<br><br>Show the field ONLY if:<br>[dispo_final] = '1' or [dispo_final] = '2' or [dispo_final] = '4' or [dispo_final] = '66'	What was the Neurologic Status at Hospital Discharge* (Glasgow Pittsburgh Classification)	radio, Required<br>1 Good<br>2 Mild Impairment<br>3 Moderate Impairment<br>4 Severe/Vegetative Impairment<br>5 Dead<br>88 Unknown<br><br>Custom alignment: LH
	27	[date_injury]	Section Header: *DEMOGRAPHICS*<br><br>Date of Injury	text (date_mdy, Min: 2016-01-01, Max: 2024-12-31), Required<br>Custom alignment: LV<br>Field Annotation: @HIDEBUTTON
	28	[dob]	Date of birth	text (date_mdy), Required, Identifier<br>Custom alignment: LV<br>Field Annotation: @HIDEBUTTON
	29	[pat_age_calc]	Age (years) - Calculated	calc, Required<br>Calculation: rounddown(datediff([dob],[date_injury],'y'))<br>Custom alignment: LV
	30	[pat_age]<br><br>Show the field ONLY if:<br>[pat_age_calc]=''	Age (years) - Enter age if dob is not available	text (integer), Required<br>Custom alignment: LH
	31	[sex]	Gender	radio, Required

				0 Female<br>1 Male<br>88 Unknown, not reported<br><br>Custom alignment: LH
	32	[ethnicity]	Ethnicity	radio, Required<br>0 Hispanic or Latino<br>1 NOT Hispanic or Latino<br>88 Unknown / Not Reported<br><br>Custom alignment: LH
	33	[race]	Race	radio, Required<br>0 American Indian/Alaska Native<br>1 Asian<br>2 Native Hawaiian or Other Pacific Islander<br>3 Black or African American<br>4 White<br>5 More Than One Race<br>88 Unknown / Not Reported<br><br>Custom alignment: LV
	34	[insurance]	What was the Insurance of the patient?	radio, Required<br>0 None<br>1 Public (Medicaid, CHIP, STAR)<br>2 Private (HMO, PPO - Blue Cross, Cigna, United Health Care, Aetna, etc.)<br>66 Other (Tricare, Department of Defense, etc.)<br>88 Unknown<br><br>Custom alignment: LV
	35	[address_of_drowning]	What is the STREET ADDRESS where the DROWNING OCCURRED?	notes
	36	[zip_injury]	What is the ZIP code where the DROWNING OCCURRED?	text (zipcode)
	37	[address_of_pt]	What is the PATIENT'S RESIDENTIAL STREET ADDRESS?	notes, Required, Identifier
	38	[zip]	Patient's residential ZIP code	text (zipcode), Required, Identifier
	39	[hospital_zip]	Treating Hospital ZIP code	text (zipcode), Required

	40	[inj_intent]	What was the Injury Intent?	radio, Required<br>1 Unintentional (Accidental)<br>2 Suicide<br>3 Homicide<br>4 Undetermined<br>88 Unknown<br><br>Custom alignment: LV
	41	[body_of_water]	Section Header: *DROWNING DETAILS*<br>What was the Body of Water where the victim drowned?	radio, Required<br>1 Swimming Pool (in ground, above ground, inflatable)<br>2 Hot Tub/Jacuzzi<br>3 Bathtub<br>4 Open Body of water (pond, lake, retention pond)<br>5 Open Body of water (ocean)<br>6 Flowing water (river, bayou, creek, canal, drainage ditch)<br>7 Bucket<br>8 Flood waters due to natural disaster<br>9 Well and water hole<br>66 Other<br>88 Unknown<br><br>Custom alignment: LV
	42	[body_water_othr]<br>Show the field ONLY if:<br>[body_of_water] = '66'	Specify other body of water where the victim drowned.	text, Required<br>Custom alignment: LV
	43	[boatrelate]<br>Show the field ONLY if:<br>[body_of_water] = '4' or [body_of_water] = '5' or [body_of_water] = '6' or [body_of_water] = '8' or [body_of_water] = '66' or [body_of_water] = '88'	Was drowning boating related?	radio, Required<br>1 Yes<br>0 No<br>88 Unknown<br><br>Custom alignment: LH
	44	[victim_activity]	What was the activity of the victim at the time of the drowning?	radio, Required<br>1 Bathing

				2 Playing<br>3 Wading<br>4 Swimming<br>5 Jumped in<br>6 Fishing<br>7 Boating (non-motorized, canoe, paddle boat)<br>8 Boating (motorized)<br>9 Towed from motorized watercraft (tubing, water skiing, surfing)<br>10 Jet ski (small watercraft)<br>11 Sailing<br>12 Diving<br>13 Surfing<br>14 Snorkeling<br>15 Driving motor vehicle into body of water<br>16 Fell in body of water<br>66 Other (not specified above)<br>88 Unknown<br><br>Custom alignment: LV
	45	[body_water_othr_2]<br><br>Show the field ONLY if:<br>[victim_activity] = '66'	Specify other activity of victim at time of the drowning.	text, Required<br>Custom alignment: LV

	46	[precip_event]	What was the precipitating event?	radio, Required<br>1 Seizure<br>2 Pushed in or dunked<br>3 Trauma<br>4 Drug Intoxication<br>5 Cardiac or Syncope<br>6 Breath holding prior to submersion<br>7 Natural disaster/flooding event<br>66 Other<br>88 Unknown<br><br>Custom alignment: LV
	47	[precip_event_othr]<br><br>Show the field ONLY if:<br>[precip_event] = '66'	Specify other precipitating event.	text, Required<br>Custom alignment: LV
	48	[water_access]	What was the victim's access to the body of water where the drowning occurred?	radio, Required<br>1 Private residential swimming pool/hot tub<br>2 Apartment or Condominium swimming pool<br>3 Subdivision or Neighborhood swimming pool<br>4 Hotel or Motel swimming pool/hot tub<br>5 Nearby bayou, stream or river<br>6 Nearby lake or ocean<br>7 Private residence bathtub<br>66 Other<br>88 Unknown<br><br>Custom alignment: LV
	49	[water_access_othr]<br><br>Show the field ONLY if:<br>[water_access] = '66'	Specify other victim's access body of water	text, Required<br>Custom alignment: LV
	50	[county_drown]	County of Drowning	radio<br>1 Harris<br>2 Montgomery

				3 Brazoria<br>4 Fort Bend<br>5 Liberty<br>6 Wharton<br>7 Austin<br>8 Chambers<br>9 Galveston<br>10 Livingston<br>11 Waller<br>12 Travis<br>66 Other<br><br>Custom alignment: LV
	51	[county_drown_othr]<br><br>Show the field ONLY if:<br>[county_drown] = '66'	Specify other county of drowning	text, Required<br>Custom alignment: LV
	52	[sub_time]	What was the duration of submersion? (Time under water)	radio, Required<br>1 0-1 minute<br>2 2-3 minutes<br>3 4-5 minutes<br>4 > 5 minutes<br>88 Unknown<br><br>Custom alignment: LH
	53	[past_medical]	Section Header: *RISK FACTORS*<br><br>Prior Medical Problems	checkbox, Required<br>1 past_medical___1 Epilepsy<br>2 past_medical___2 Autism or Developmental delay<br>3 past_medical___3 Cardiac (Heart Disease)<br>4 past_medical___4 Behavioral (Depression, Bipolar, Anxiety Disorder)<br>5 past_medical___5 Respiratory (Asthma, COPD)

			6 past_medical___6 Neuromuscular or joint problems<br>66 past_medical___66 Other<br>0 past_medical___0 None<br>88 past_medical___88 Unknown<br><br>Custom alignment: LV<br>Field Annotation: @NONEOFTHEABOVE='0,88'
54	[past_medical_othr]<br><br>Show the field ONLY if:<br>[past_medical(66)] = '1'	Specify other medical problems	text, Required<br>Custom alignment: LV
55	[drug_intake]	Did the victim consume ALCOHOL/DRUGS at the time of drowning (based on history or drug test)?	radio, Required<br>1 yes<br>0 No<br>88 Unknown<br><br>Custom alignment: LH
56	[drugs]<br><br>Show the field ONLY if:<br>[drug_intake] = '1'	What drug(s) did the drowning victim consume?	checkbox, Required<br>1 drugs___1 Alcohol<br>2 drugs___2 Marijuana<br>3 drugs___3 Nicotine<br>4 drugs___4 Opioids<br>5 drugs___5 Sedatives (Diazepam, barbiturates)<br>66 drugs___66 Other<br>88 drugs___88 Unknown<br><br>Custom alignment: LV<br>Field Annotation: @NONEOFTHEABOVE='88'
57	[drug_intake_othr]<br><br>Show the field ONLY if:<br>[drugs(66)] = '1'	Specify 'other' drug(s) the drowning victim consumed	text, Required<br>Custom alignment: LV
58	[swim_know]	Section Header: *WATER COMPETENCY*<br><br>Did the victim know how to swim?	radio, Required<br>1 Yes

				0 No (documented in medical chart)<br>2 Unsure (floaties used in swimming pool)<br>88 Unknown<br><br>Custom alignment: LH
	59	[water_head]<br><br>Show the field ONLY if:<br>[pat_age] < 18 or [pat _age_calc] < 18	Was the child comfortable playing with water over his/her head?	radio, Required<br>1 Yes<br>0 No<br>88 Unknown<br><br>Custom alignment: LH
	60	[fence]<br><br>Show the field ONLY if:<br>[body_of_water] = '1' or [body_of_water] = '2'	Section Header: *PROTECTIVE FACTORS*<br><br>If the drowning was in a SWIMMING POOL/HOT TUB was the pool FENCED?	radio, Required<br>1 Yes<br>0 No<br>88 Unknown<br><br>Custom alignment: LH
	61	[fencing_type]<br><br>Show the field ONLY if:<br>[fence] = '1'	If swimming pool/hot tub was FENCED, was the fencing:	radio, Required<br>1 Four-sided<br>2 Three-sided with the house being on one side of the pool<br>66 Other<br>88 Unknown<br><br>Custom alignment: LH
	62	[fence_type_othr]<br><br>Show the field ONLY if:<br>[fencing_type]=66	Specify 'other' swimming pool/hot tub was FENCED, fencing type	text, Required<br>Custom alignment: LV
	63	[door_alarm]<br><br>Show the field ONLY if:<br>[fence] = '1'	If swimming pool/hot tub was fenced on 4 sides or fenced on 3 sides with the house making up the remaining side, was there a functioning DOOR ALARM on the door that had access to the pool?	radio, Required<br>1 Yes<br>0 No<br>88 Unknown<br><br>Custom alignment: LH
	64	[gate]<br><br>Show the field ONLY if:<br>[fence] = '1'	If swimming pool/hot tub was fenced, did the fence have a FUNCTIONING, SELF CLOSING, SELF LATCHING GATE?:	radio, Required<br>1 Yes<br>0 No<br>66 Other

				88 Unknown<br><br>Custom alignment: LH
	65	[gate_othr]<br><br>Show the field ONLY if:<br>[gate]=66	Specify 'other' swimming pool/hot tub was fenced, did the fence have a FUNCTIONING, SELF CLOSING, SELF LATCHING GATE?	text, Required<br>Custom alignment: LV
	66	[doorunlock]<br><br>Show the field ONLY if:<br>[body_of_water] = '1' or [body_of_water] = '2'	Was the door that has access to pool/hot tub left open or unlocked?	radio, Required<br>1 Yes<br>0 No<br>99 Unknown<br><br>Custom alignment: LH
	67	[lifejacket]	What PROTECTIVE DEVICE was the victim wearing at the time of drowning?	radio, Required<br>0 None<br>1 Life Jacket<br>3 Puddle Jumper<br>66 Other<br>77 Not Applicable<br>88 Unknown<br><br>Custom alignment: LH
	68	[lifejacket_othr]<br><br>Show the field ONLY if:<br>[lifejacket]=66	Specify 'other' PROTECTIVE DEVICE was the victim wearing at the time of drowning	text, Required<br>Custom alignment: LV
	69	[lifeguard]	Was the victim rescued by a LIFEGUARD?	radio, Required<br>1 Yes<br>0 No<br>88 Unknown<br><br>Custom alignment: LH
	70	[boat_status]<br><br>Show the field ONLY if:<br>[boatrelate]=1	Section Header: *BOATING FACTORS AND OPEN WATERS*<br><br>What was the victim's role on the boat?	radio, Required<br>1 Passenger<br>2 Operator<br>66 Other<br>88 Unknown<br><br>Custom alignment: LH
	71	[boat_status_othr]	Specify 'other' victim's role on the boat	text, Required<br>Custom alignment: LV

		Show the field ONLY if:<br>[boat_status]=66		
	72	[witnessed]	Section Header: *RESUSCITATION*<br>Was the drowning WITNESSED? (The act of submersion was witnessed)	radio, Required<br>1 Yes<br>0 No<br>88 Unknown<br>Custom alignment: LH
	73	[rescuer]	Who RESCUED the victim?	radio, Required<br>1 Parent (includes step parents)<br>2 Adult relative (includes adult sibling)<br>3 Underage person (includes sibling or other person < 18 years of age<br>4 Lifeguard<br>66 Other Adult person (includes baby sitter, friend)<br>88 Unknown<br>Custom alignment: LV
	74	[resus]	Did the drowning victim receive RESUSCITATION?	radio, Required<br>1 Yes<br>0 No<br>88 Unknown<br>Custom alignment: LV
	75	[who_resus]<br>Show the field ONLY if:<br>[resus]=1	Who administered the RESUSCITATION?	radio, Required<br>1 Bystander (any person other than pre-hospital medic)<br>2 EMS<br>66 Other person<br>88 Unknown<br>Custom alignment: LH
	76	[other_resus]<br>Show the field ONLY if:<br>[who_resus] = '66'	Specify 'Other Person" resuscitation	text, Required<br>Custom alignment: LH
	77	[resus_type]	If the drowning victim received resuscitation, what was the TYPE OF	checkbox, Required

		Show the field ONLY if:<br>[resus] = '1'	RESUSCITATION administered?	1 | resus_type___1 | Chest compressions<br>2 | resus_type___2 | Rescue breaths<br>3 | resus_type___3 | CPR (as mentioned in the records)\\<br>4 | resus_type___4 | Back blows<br>5 | resus_type___5 | Heimlich Maneuver<br>6 | resus_type___6 | AED (Automatic External Defibrillator)<br>66 | resus_type___66 | Other<br>88 | resus_type___88 | Unknown<br><br>Custom alignment: LV<br>Field Annotation: @NONEOFTHEABOVE='88'
	78	[resus_type_othr]<br><br>Show the field ONLY if:<br>[resus_type(66)] = '1'	Specify other resuscitation type	text, Required<br>Custom alignment: LV
	79	[supervision]	Section Header: *SUPERVISION*<br><br>Was the drowning victim being SUPERVISED?	radio, Required<br>1 | Yes<br>0 | No<br>77 | Not applicable<br>88 | Unknown<br><br>Custom alignment: LH
	80	[supervisor]<br><br>Show the field ONLY if:<br>[supervision] = '1'	Who was SUPERVISING the victim?	radio, Required<br>1 | Parent (includes step parents)<br>2 | Adult relative (includes adult sibling)<br>3 | Underage person (includes sibling or other person < 18 years of age<br>66 | Other Adult person (includes baby sitter, friend)<br>88 | Unknown<br><br>Custom alignment: LV

	81	[supervision_type]<br>Show the field ONLY if:<br>[supervision] = '1'	If the drowning victim was being supervised, what was the LEVEL of SUPERVISION?	radio, Required<br>1 Within close proximity (touch) and engaged<br>2 Within vicinity and visual<br>3 Unsupervised<br>88 Unknown<br>Custom alignment: LH
	82	[demographics_and_submersion_details_complete]	Section Header: *Form Status*<br>Complete?	dropdown<br>0 Incomplete<br>1 Unverified<br>2 Complete
